# The AC-OK Cooccurring Screen: Reliability, Convergent Validity, Sensitivity, and Specificity

**DOI:** 10.1155/2013/573906

**Published:** 2012-12-23

**Authors:** Andrew L. Cherry, Mary E. Dillon

**Affiliations:** ^1^School of Social Work, University of Oklahoma, Tulsa Campus, Tulsa, OK 74135, USA; ^2^University of Central Florida, Orlando, FL 32816, USA

## Abstract

The principal barriers to universal screening for the cooccurring disorders of mental illness and substance abuse are training, time, cost, and a reliable and valid screen. Although many of the barriers to universal screening still remain intact, the lack of a cooccurring screen that is effective and can be administered in a cost efficient way is no longer an obstacle. This study examined the reliability, factor structure, and convergent validity of the 15-item AC-OK Cooccurring Screen. A total of 2,968 AC-OK Cooccurring Screens administrated to individuals who called or went to one of the nine participating mental health and substance abuse treatment facilities were administrated and analyzed. Principal axis factor (PAF) analysis was used in the confirmatory factor analysis to identify the common variance among the items in the scales while excluding unique variance. Cronbach's Alpha was used to establish internal consistency (reliability) of each subscale. Finally, the findings from the AC-OK Cooccurring Screen were compared to individual scores on two standardized reference measures, the addiction severity index and the Client assessment record (a measure of mental health status) to determine sensitivity and specificity. This analysis of the AC-OK Cooccurring Screen found the subscales to have excellent reliability, very good convergent validity, excellent sensitivity, and sufficient specificity to be highly useful in screening for cooccurring disorders in behavioral health settings. In this study, the AC-OK Cooccurring Screen had a Cronbach's Alpha of .92 on the substance abuse subscale and a Cronbach's Alpha of .80 on the mental health subscale.

## 1. Introduction

The knowledge that the cooccurring disorders of addiction or mental illness are widespread in the treatment population and are more complicated to treat has long been the accepted reality among practitioners and researchers. These assumptions are well founded. Estimates based on the 2009 National Survey on Drug Use and Health in the United States suggest that among the 45.1 million adults 18 years of age or older who experienced a mental illness in the year before the survey 8.9 million adults (19.7%) met criteria for substance dependence or abuse (a cooccurring disorder) in that period compared with 6.5% (11.9 million adults) who did not have mental illness in the past year. Based on these numbers, an important finding for alcohol and drug treatment programs is that among the 20.8 million adults who were identified with a substance use disorder, 4 out of 10 (43%) will also have a mental illness that will need to be treated. 

Currently, the recommended approach for treating a person with a cooccurring disorder is an Integrated Treatment model. The objective is to treat both disorders simultaneously. Although identified as an evidence-based treatment by SAMHSA, this does not mean that parallel or sequential treatment is less effective. At this point in time, the results of randomized controlled trials to determine the effectiveness of the Integrated Treatment model are “equivocal but encouraging” [1]. Even so, program administrators have recognized the disadvantages of separate treatment systems and are attempting to increase integrative approaches [2]. Above all, integrated treatment is designed to increase effectiveness by minimizing obstacles experienced by individuals who are seeking care for cooccurring disorders [3]. In a context where there is a need to identify people with a cooccurring disorder, for example, by programs exclusively treating addiction and programs exclusively treating mental health disorders, a screen for cooccurring would also contribute to more attention being paid to both disorders. 

## 2. The Need for an Integrated Screening Tool

 A formidable challenge to implementing treatment for people with a cooccurring disorder is the lack of a practical way of screening people for this comorbid disorder, as Lehman [4] pointed out the lack of screening and assessment for cooccurring disorders was an important barrier that prevented less than effective treatment reaching people with a cooccurring disorder. One approach that can be used to identify people with a cooccurring disorder is to conduct two assessments (one to identify a substance use disorder and one to detect a mental health disorder) on everyone who asks for help with a behavioral health disorder [5]. Although effective, this is an expensive strategy. Training staff to administer a large battery of tests, the time involved in administering and interpreting multiple assessments, and the lack of funders who are willing to pay for a large battery of assessments on everybody that seeks treatment make this approach cost prohibitive for most agencies. Given these realities, an *integrated screening tool* that will identify people who likely have a cooccurring disorder is sorely needed. An *integrated screening tool* is defined as a brief measure that can identify both an addition and mental health problem. An *integrated screening tool *is not intended to assess or diagnose, or determine the severity of the disorder, “but only whether or not the person is likely to have a disorder and indicates when additional assessment is needed” [6]. 

Consequently, rather than completing two assessments on each person who seeks behavioral health services, it is more cost effective to use a rapidly administered screen to identify people who are most likely to have a cooccurring disorder and then do an assessment on only those individuals [6, 7]. 

The literature search conducted to identify screening instruments found journals and monographs from the United States, Canada, Australia, and England that addressed issues of screening and assessing for a cooccurring disorder. A monograph published by the Population Health Division, Australian Government Department of Health and Ageing, provided a detailed review for usable measures and screens related to substance abuse and mental disorders. It covers the measures described in a matrix and several others used internationally [8]. Another monograph was the* Best Practices: Concurrent Mental Health and Substance Use Disorders *[9]. As well, the monograph, TIP 42, *Substance Abuse Treatment for Persons with Cooccurring Disorders *also reviews instruments available for screening and assessment [5].

Among the scales found in the literature that could be used as a screen, the majority were time consuming to administer and nearly all required specific clinical skills and training and covered only one disorder (i.e., mental health, or substance abuse). While there are few scales specifically designed to identify people with a cooccurring disorder, the Dartmouth Assessment of Lifestyle Instrument (DALI) is a notable exception [10]. The DALI is a substance abuse measure in the public domain that has 18 questions. It is specifically designed to detect problematic alcohol and marijuana use among people who are *inpatient at a mental health facility*. It does not address mental health issues. The DALI uses weighted items that must be scored. Unlike the AC-OK Cooccurring Screen (AC, Andrew Cherry; OK, Oklahoma), some training is needed to effectively administer this measure.

The CAGE-AID is another instrument that is being used in the mental health field to identify people with a mental health disorder that have a substance use problem [11]. Although not designed to screen for both mental illness and drug use, the CAGE-AID was validated with a sample of people with severe mental illness. In this case, the CAGE-AID performed better than other approaches such as clinical variables, laboratory tests, and collateral reports. A serious limitation, however, is that the CAGE-AID collects information related to *lifetime *rather than *current *substance use problems. This tends to be a limitation when the purpose of the screen is to determine the need for an assessment to determine a cooccurring disorder. In contrast, the AC-OK Cooccurring Screen can be rapidly administered, is easy to interpret, and takes little or no training to administer.

The Comprehensive Addictions and Psychological Evaluation (CAAPE) is another comprehensive diagnostic assessment interview that assesses for substance misuse and mental health disorders designed to assess for cooccurring disorders. The criteria for each diagnosis are based on the DSM-IV TR. This is a fairly extensive questionnaire that covers 14 mental health and substance abuse conditions [12]. 

The need to screen for a cooccurring disorder is strongly supported by the literature. Yet, there are no integrated screening tools available that are specifically designed to identify people who are likely to have a cooccurring disorder of mental illness and substance abuse. The AC-OK Cooccurring Screen was designed to meet that need. The following provides the psychometric information from the analysis of the AC-OK Cooccurring Screen.

## 3. Development of the AC-OK Cooccurring Screen

The AC-OK Cooccurring Screen is a version of an earlier screen described in more detail elsewhere [13]. This was a pilot study with 200 volunteer participants. The results of the pilot showed very good psychometric statistics for the items and structure, and provided valuable data that was used to refine the version presented here, the AC-OK Cooccurring Screen. This screen was designed to be a “hot button screen.” Hypothetically, if a person asking for treatment services answers “yes” to at least one question in both the mental health and substance abuse subscales, that person would then be administered an assessment battery to determine if the person has a cooccurring disorder. 

The original set of questions for the screen came from an advisory group consisting of experts in psychometrics (instrument construction), professionals in both private and public practice from the fields of mental health, and substance abuse. Additionally, people who represented advocacy groups and service recipients were on the committee. 

The AC-OK Cooccurring Screen identifies four broad spectrum disorders listed in the DSM-IV TR (i.e., substance use, psychoses, mood disorders, and anxiety spectrum Disorder). The AC-OK Cooccurring Screen is designed to collect self-report information from people seeking either mental health or substance abuse treatment services. Self-report information on substance abuse and mental health is typically considered reliable and valid unless the context in which the questions are asked has potential negative consequences for the person such as loss of job, arrest, and hospitalization [14]. 

## 4. Methodology

The definition of a person with a cooccurring disorder that underpins the AC-OK Cooccurring Screen is: “…individuals who have at least one psychiatric disorder as well as an alcohol or drug use disorder. While these disorders may interact differently in any one person (e.g., an episode of depression may trigger a relapse into alcohol abuse, or cocaine use may exacerbate schizophrenic symptoms) at least one disorder of each type can be diagnosed independently of the other” [15]. 

After IRB approval was received from both the researcher's University and the State Agency of Mental Health and Substance Abuse Treatment, data collection was carried out over a six-month period by nine different agencies. Limited data was solicited from people not admitted for treatment to be in compliance with the Health Insurance Portability and Accountability Act (HIPAA) rules and to protect the identity of any person inquiring about treatment services. 

## 5. Participants

Those who participated in the pilot of the AC-OK Cooccurring Screen were individuals who called or went to one of nine southwestern behavioral health agencies seeking help for what they believed was a mental health or a substance misuse problem. The AC-OK Cooccurring Screen was integrated into the intake process at the nine agencies. Five agencies specialized in mental health treatment and four agencies specialized in addiction treatment. There were a total of 2,968 screens completed. Of those, 1,714 people (58%) who completed the screen entered a treatment facility. For people who did not enter treatment, there is no information beyond the individual's responses to the screen, and the agency that did the screening. There were 2,267 screens completed at the mental health agencies, and 701 screens completed at the substance abuse treatment centers.

These participants were not a homogeneous group. The heterogeneity, however, is not a result of a failure of the sampling technique or the sample drawn, but is a natural reflection of the people seeking help from these behavioral health agencies (no selection bias present). The nine agencies that participated in this study were selected (based on statewide treatment data) because they represented both urban and rural treatment populations. Based on the following analysis and the large number of people screened, the results of the analysis should be strongly reflective of a large group of people seeking help for a mental health, a substance abuse, or a cooccurring disorder.

### 5.1. Age, Gender, and Those Admitted and Not Admitted

The average age of people seeking treatment in this population was 35.7 years with a standard deviation of 10.87 years. The participants ranged in age from 18 to 75 years old. Some 50% were between 18 and 35 years of age. Of those who completed the screen, 56.3% were male and 43.7% were females.

### 5.2. Data Collected on Those Admitted and Not Admitted

The only data collected on people asking for help and who were not admitted were gender, age, and agency where the screen was completed, and the person's responses to the screen questions. If the person screened was later admitted to a treatment program additional deidentified admission information was added to the screen information collected on the individual. 

### 5.3. Comparing People Not Admitted to People Who Were Admitted on Age and Gender

When those *not* admitted to treatment are compared to those who were admitted to treatment, the average age for both groups is virtually the same. The average age of those* not* admitted was 35.87 years with a standard deviation of 11.08 years. They ranged in age from 18 to 75 years of age. The average age for those admitted was 35.52 years with a standard deviation of 10.63 years. They ranged in age from 18 to 72 years of age. By gender among those *not* admitted, 56.8% were males and 43.2% were females. Among those admitted, 55.9% were males and 44.1% were females. 

They were no significant differences between age and gender among people in the two groups. Age was tested using the *t*-test (*t* = −.958, df = 3593.44, *P* < .339). Gender differences were tested using the chi-square test for significance (*χ* ^2^ = .247, df = 1, *P* < .601).

Based on the data gathered from this population of people who called or went to a mental health or substance abuse treatment agency for service during this study period, statistical tests were used to determine the reliable, convergent validity, and discriminate validity, sensitivity, and specificity of the two screen subscales. The overarching aim of the analysis was to determine how well the AC-OK Cooccurring Screen subscales can be expected to screen out people with only one disorder and screen in people who possibly have both disorders. 

The first step in the analysis was to do a confirmatory factor analysis. Confirmatory factor analysis is a theory-testing approach. Confirmatory factor analysis begins with a hypothesis that specifies which items will be correlated with which factors [16]. In this study, confirmatory factor analysis was used to verify or invalidate the construct of the 15 questions that were hypothesized to form two dimensions; one of mental illness items and one of substance abuse items. 

The preferred extraction method when conducting a confirmatory factor analysis is Principal Axis Factor (PAF) analysis, because this method identifies the common variance among the variables, excluding unique variance. Appropriate factor loadings above  .50 are defined as supporting the construct validity of the scale.

The second step employed Cronbach's Alpha to measure the internal consistency (reliability) of each of the two subscales. Finally, the AC-OK Cooccurring Screen was compared to two standardized references the Addiction Severity Index (ASI) and the Client Assessment Record (CAR). 

The ASI was designed to gather information from people who request alcohol or drug abuse treatment on seven areas of life: medical, employment/support, drug and alcohol use, legal, family history, family/social relationships, and psychiatric problems. The Client Assessment Record (CAR) is a clinical assessment of problems, strengths, and functioning related to one's mental health. The CAR is designed to provide the clinician with a comprehensive overview of an individual's capacity to function in the community. It measures depression, anxiety, mania, attention, thought, disrespect, issues, suicide, self-care, dangerousness, security, family, role, issues, substance, and legal on six (6) levels of functioning. The ASI and CAR are used by the agencies to determine the severity of addiction and mental illness and the need to be admitted for treatment.

## 6. Results

Principal Axis Factoring (PAF) was used to confirm the association among items in the subscales. First, the screen data was converted to a tetrachoric correlation matrix [17]. In this analysis, tetrachoric correlation coefficients are used because the responses on the screen items are binary (yes, 1; no, 2). A tetrachoric correlation between items with binary responses estimates the correlation as if the two responses (“yes” and “no”) were continuous measures [18]. In this analysis the tetrachoric correlations are considered a special case of latent trait modeling. As a result, an analysis of a tetrachoric correlation tends to give a more precise view of the latent structure that underlies the response characteristics of a scale using items with binary responses [19–21].

Next, to evaluate the appropriateness of using a factor analysis procedure with this matrix of tetrachoric correlations, the Bartlett's Test of Sphericity was used. This test suggests that the data met the basic assumption of sphericity (*χ* ^2^ = 52040.98, df = 105, *P* < .000). In other words, the correlations in the data matrix are not due to sampling error. 

The Kaiser Measure of Sample Adequacy (KMO) was the second test used to check the appropriateness for employing a factor analysis procedure with this data. The resulting KMO for the mental health scale was  .914. Kaiser considered KMO's in the  .80's as “meritorious” [22]. These tests suggest that the data are adequate for using in a PAF Analysis. 

Once it was established that the data from the screen items could be used in a factorial procedure, the structure of the tetrachoric correlation matrix was investigated using PAF as the guiding extraction criteria. In this analysis, the screen items with factor loadings of  .50 were selected because they are more likely to produce stable and reliable subscales [23–25]. The PAF analysis extracted two factors.  Table 1 presents the Varimax rotated factor loadings. The two-factor solution accounted for 65.65% of the variance. The factor analysis in Table 1 shows that the questions from the screen make up two clear and compelling dimensions, mental illness, and substance abuse.

## 7. Reliability Analyses 

Once the items in each domain were confirmed by the PAF analysis, the Cronbach's Alpha coefficient was used to determine the degree of response consistency among those screened who answered the items on the two subscales. Using the items in the two subscales (the set of questions developed by committee and the same questions identified in the two factors produced by the PAF analysis) a Cronbach's Alpha coefficient was computed for each of the two subscales. The results of these analyses are presented in Table 2.

For the nine items that make up the mental health screen, the mean score was 12.82 with a standard deviation of 2.62 with a 95% confidence interval ranging from 12.74 to 12.90. The median score was 12 (mode = 11) with scores ranging from 9 to 18. Finally, the distribution of scores was slightly skewed (0.399; SE = 0.41) with a kurtosis of −0.80 (SE = 0.82). An examination of the corrected item-total correlations suggests that all nine items are moderately correlated to the total score. In the analysis, corrected item-total correlations range from a low of  .42 to a high of  .55 with a final Alpha of  .795. Moreover, if any item is removed from the scale the Cronbach's Alpha Coefficient will be reduced. 

For the six items reflecting the substance abuse subscale, the mean score is 8.69 with a standard deviation of 2.37 with a 95% confidence interval ranging from 8.61 to 8.76. The median score was 8.00 (mode = 6.00) with scores ranging from 6.00 to 12.00. Finally, the distribution of scores was not skewed (0.19; SE = 0.04) with a kurtosis of −1.61 (SE = 0.08). Examination of the corrected item-total correlations among the substance abuse items suggests that all six items are strongly correlated to the total score. In this analysis, corrected item-total correlations range from  .73 to  .81. The final Cronbach's Alpha coefficient was  .922. If any item is removed from the scale the Cronbach's Alpha Coefficient will be reduced. 

Given the results of both the PFA and item analysis of the screen's two subscales, the psychometric stability of the screen is reasonably positive, indicating a moderate to high level of reliability. The substance abuse screen has excellent reliability (*α* = .92). The mental health screens has good reliability (*α* = .80). Test-retest reliability has not been established. The client data had to be deidentified per HIPPA requirements, which made it impossible to use test-retest methodology in this study.

## 8. Convergent and Discriminant Validity

After finding that the two subscales in the AC-OK Cooccurring Screen had excellent to good reliability, the validity of the AC-OK Cooccurring Screen was tested. Pearson correlation coefficients were used to test the levels of convergent and discriminant validity. In this analysis, the two subscales of the screen are correlated with the reference scales (i.e., the Addiction Severity Index and the Client Assessment Record). 

The substance abuse treatment agencies that participated in this study were required by state law to use the Addiction Severity Index (ASI) to determine if an individual should be admitted for treatment. The ASI uses a 10-point scale to rate severity based on historical and current information. It is designed to be administered in a semistructured interview that takes approximately an hour [26]. The reliability alpha for the ASI ranges from  .56 to  .85. The ASI-psych is a subscale of the ASI that gathers information and severity of psychiatric problems. The ASI-psych reliability alpha ranges in the low  .90s. The ASI psychometrics with substance abuse populations have been well established over many years. The primary focus of the ASI is on substance use. However, it also contains a section on psychological problems and functioning. Extensive training materials are available for the ASI. It takes a relatively low level of skill to administer. It is easily scored and relatively inexpensive. Trained lay interviewers are needed to administer the ASI [27]. The subscale of the ASI that measures the severity of psychopathology (ASI-psy) is used as a reference scale to the measure of mental health. 

The CAR scales are not designed to be administered as standalone scales. Their reliability depends in part on being embedded in the CAR assessment interview. Reliabilities across CAR scales are reported to ranging from  .67 to  .87. The CAR-substance use scale reliability alpha ranges from  .70 to  .80. The clinician needs to be trained in the administration of the CAR. Typically, the information needed to complete the CAR is gathered by the clinician from a face-to-face semiinterview with the client [28]. The subscale of the CAR, the substance use scale (CAR-sa), measures the extent to which a person used addictive drugs. The CAR-sa is used as a reference scale to measure alcohol and other drug abuse.

The correlations between the reference scales, the two AC-OK Cooccurring Screen subscales and the ASI-psy and the CAR-sa are presented in Table 3. As expected, a substantial and significant correlation was found between the AC-OK Cooccurring Screen mental health measure and the ASI-psy (*r* = .56; *P* < .001). Additionally, a substantial and significant correlation was found between the AC-OK Cooccurring Screen substance abuse measure and the CAR-sa (*r* = .59; *P* < .001). These correlations represent concurrent validity between the AC-OK Screen and the ASI-psy and the CAR-sa. The correlations (interpreted like any Pearson correlation of this magnitude) show a moderate level of convergent validity providing support that the AC-OK Screen is a reliable and valid brief screen for detecting possible co-occurring disorders.

## 9. Screen Sensitivity and Specificity

The AC-OK Cooccurring Screen was designed to be used in behavioral health settings. This is important because in a setting where a large number of people are expected to have a cooccurring disorder (i.e., mental health and substance abuse treatment settings), the goal is to miss as few people with the disorder as possible. In these settings, the *sensitivity* of a screen is stressed. On the other hand, where a small number of people with a cooccurring disorder are expected to request services (i.e., a general medical practice setting) the goal is to identify as few false positives as possible. In these settings *specificity* is stressed. 

The purpose of the AC-OK Cooccurring Screen is twofold. Its primary purpose is to identify people that may have a cooccurring disorder (sensitivity). Secondly, it is designed to identify people who most likely do *not* have cooccurring disorder, people who do *not* need a comprehensive assessment for a cooccurring disorder (specificity).

*Sensitivity* is defined as the probability of a screen testing positive, when in fact the person being screened has the disorder (the true positive rate). Likewise, high sensitivity suggests that a negative screen score indicates that an assessment is not needed. 

*Specificity* is defined as the probability that a negative screen test is negative because the person does not have the disorder (the true negative rate). Conversely, high specificity suggests that a positive screen score strongly suggests that an assessment is needed. Overall accuracy of the screen is determined by the combination of sensitivity and specificity [6]. 

The AC-OK Cooccurring Screen based on this group of participants had a higher level of *sensitivity* than *specificity*. As a result, the AC-OK Cooccurring Screen produced a fair number of false positives (people identified by the Screen who did not have a cooccurring disorder). This is acceptable, however, because in mental health and substance abuse treatment agencies, the goal is to miss as few people as possible with a cooccurring disorder. 

The sensitivity of the AC-OK Cooccurring Screen is excellent. It missed only 7 out of 177 people (96% correctly identified) who were later found to have a cooccurring disorder using the ASI-psy. Among people assessed with the CAR-sa and found to have a cooccurring disorder, the Screen missed 48 out of 507 people (90.5% correctly identified) (see Table 4). 

The new Screen's specificity is fair. The Screen identified approximately 72% of all people screened as needing a comprehensive assessment to determine if the person had a cooccurring disorder. The number of people in this population with a cooccurring disorder was estimated to be 35% [29]. This lower level of specificity suggests that about 50% of the people identified as having a potential cooccurring disorder by the AC-OK Cooccurring Screen will actually have the disorder (see Table 4). The level of specificity could be improved but when specificity increases sensitivity decreases. 

## 10. Discussion

The data collected to determine the reliability, convergent and discriminant validity, sensitivity, and specificity of the AC-OK Cooccurring Screen strongly suggests that the psychometric stability of the screen is reasonably positive, indicating a moderate to high level of reliability. The substance abuse screen has the best reliability (*α* = .92). The mental health screen has good reliability (*α* = .80). In terms of *sensitivity*, the AC-OK Cooccurring Screen can be expected to identify about 70% of those being screened as needing to be assessed for a cooccurring disorder. Of this number, the AC-OK Cooccurring Screen missed about 7% of people who could be identified as having a cooccurring disorder if they were fully assessed for both a mental health and substance use disorder.

One limitation of the analysis is that AC-OK COD was not compared to the SCID (Structured Clinical Interview for DSM-IV Axis I Disorders), the gold standard for establishing specificity and sensitivity diagnoses such as a cooccurring disorder. Instead it was compared to two assessments measures used by the treatment facilities to determine severity and need for admission to treatment. Furthermore, another limitation might be the lack of strong correlations between the AC-OK COD and the two-reference scale. The lack of strong correlations in this case, however, was not because of the predictive value of the screen but because the purpose of the screen and the *two reference measures* are very different. The screen is used to detect the presence of a cooccurring disorder while the ASI-psy and the CAR-sa are used to detect the present of a disorder and to determine the severity. Another reason is the time it takes to administer two assessment tools. While psychosocial assessments like the ASI (for addiction) and the CAR (for mental health disorders) take an hour each to administer, the AC-OK Screen which takes less than five minutes to administer and score will still add 10 to 15 minutes to each client intake. For some programs this will be a financial burden. 

An issue to keep in mind when using this screen (and for that matter other screens and assessments) especially for people presenting with substance abuse problems is that some symptoms (i.e., hallucinations, paranoia, and depression) caused by intoxication or withdrawal can subside or disappear after a period of abstinence. 

Nevertheless, Authors of [30] suggested that failing to identify substance use and cooccurring mental health disorders can result in a misdiagnose and less than effective pharmacological interventions, neglect of appropriate substance abuse treatments, and inappropriate treatment planning and referral. Indeed, a best practice for providing services to people with a cooccurring disorder includes time sensitive screening of all people seeking help for a mental health or substance abuse disorder.

Screening for the single disorders such as substance abuse, mental illness, and trauma has a rich history of success. The AC-OK Cooccurring Screen is a screening tool with 15 questions that can efficiently identify people who are likely to have a cooccurring disorder. It can be easily incorporated into an agency's intake process and can be completed in five minutes or less. Moreover, the screen is easy to score. A “yes” answer on any question in the related subscales would signal the need for an assessment in that area. Based on the results of this study, the AC-OK Cooccurring Screen is reliable and valid and has high levels of sensitivity and specificity. 

The AC-OK Cooccurring Screen could also have potential to identify people who need to be assessed for a possible cooccurring disorder whether the person presents at a mental health facility or at a substance abuse treatment facility. It could also be helpful at a shelter for victims of domestic violence, in jails and detention settings, in medical clinics, in emergency rooms, and in other settings to determine if a person needed to be fully assessed for a cooccurring disorder.

Although many of the barriers to universal screening for a cooccurring disorder are still intact (training, time, cost, and an infrastructure where everybody seeking mental health or substance abuse services is screened), the lack of a cooccurring screen that is effective and can be administered economically for detecting people who need a comprehensive assessment for a cooccurring disorder is no longer a barrier.

## Questions Used in the AC-OK Cooccurring Screen

1. Have you been preoccupied with drinking alcohol and/or using other drugs?
2. Have you experienced problems caused by drinking alcohol and/or using other drugs, and kept using alcohol and/or other drugs?
3. Have you experienced serious depression (felt sadness, hopelessness, loss of interest, change in appetite, change in sleep pattern, or difficulty going about your daily activities)?
4. Have you experienced thoughts of harming yourself?
5. Have you ever been hit, slapped, kicked, emotionally or sexually hurt, or threatened by someone?
6. Have you drunk alcohol and/or used other drugs more than you intended?
7. Have you experienced a period of time when your thinking speeds up and you have trouble keeping up with your thoughts?
8. Have you experienced a traumatic event and since had repeated nightmares/dreams and/or anxiety which interferes with you leading a normal life?
9. Have you needed to drink more alcohol and/or use more drugs to get the same effect you used to get with a less amount?
10. Have you attempted suicide?
11. Have you had periods of time where you felt that you could not trust family or friends.
12. Have you been prescribed medication for any psychological or emotional problem?
13. Have you drank alcohol and/or used other drugs to alter the way you feel?
14. Have you experienced hallucinations (heard or seen things others do not hear or see)?
15. Have you tried to stop drinking alcohol and/or using other drugs, but could not?

## Figures and Tables

**Table 1:** Varimax rotated solution with factorial loadings and percentage of variance for the AC-OK Co-occurring screen (*N* = 2,969).

Items	Factor I	Factor II
SA2	**.943**	
SA1	**.920**	
SA6	**.920**	
SA9	**.915**	
SA15	**.913**	
SA13	**.882**	

MH3		**.867**
MH4		**.742**
MH7		**.732**
MH8		**.706**
MH10		**.668**
MH11		**.656**
MH12		**.650**
MH14		**.618**
MH5		**.559**
Rotated sum of squared loading: percentage of variance	35.218	30.431

*The bold numbers indicate that the associated questions are in a factor representing one of two subscales: mental health and substance abuse.

**Table 2:** Descriptive statistics and item analysis for the 15-item AC-OK Co-occurring screen (*N* = 2,968).

Item	*M*	SD	Corrected Item correlation	Alpha if Deleted
SA2	1.46	0.50	.79	.85
SA1	1.45	0.50	.76	.86
SA6	1.39	0.49	.76	.86
SA9	1.52	0.50	.76	.86
SA15	1.50	0.50	.76	.86
SA13	1.36	0.48	.39	.91

MH3	1.18	0.39	.55	.77
MH4	1.49	0.50	.53	.77
MH7	1.32	0.47	.53	.77
MH8	1.44	0.50	.52	.77
MH10	1.67	0.47	.45	.78
MH 11	1.30	0.46	.48	.77
MH12	1.36	0.48	.44	.78
MH14	1.61	0.49	.43	.78
MH5	1.46	0.50	.41	.78

Note: alphas for mental health = .922; substance abuse screen = .795.

**Table 3:** Correlations between AC-OK Co-occurring Screen and the reference scales.

	CAR SA	ASI Psy
AC-OK COD-MH	.18**	.56**
	*n* = 1,275	*n* = 525

AC-OK COD-SA	.59**	.28**
	*n* = 1,275	*n* = 525

**Correlation is significant at the 0.01 level (2 tailed).

**Table 4:** Specificity and sensitivity AC-OK Co-occurring Screen agreement compared to two reference measures.

Specificity		
The probability of the screen being positive when the person has the disorder		
AC-OK Co-occurring Screen	ASI-psy	CAR-sa
Correct	170/96%	459/90.5%
Missed	7/4%	48/9.5%

Sensitivity		
The probability of the screen being negative when the person does *not* have the disorder.		

(i) The AC-OK Co-occurring Screen identified approximately 72% of all people screened as needing an assessment to determine if the person had a co-occurring disorder.		
(ii) The estimated number of people in this population needing treatment for a co-occurring was 35%.		
(iii) The disadvantage of a lower level of sensitivity is that only half of the people being screened will be assessed as having a co-occurring disorder.		
